# The Cognitive Restoration Effects of Resveratrol: Insight Molecular through Behavioral Studies in Various Cognitive Impairment Models

**DOI:** 10.1021/acsptsci.4c00373

**Published:** 2024-10-10

**Authors:** Yingrak Boondam, Chaianan Saefoong, Natjanan Niltup, Arnaud Monteil, Worawan Kitphati

**Affiliations:** † Department of Physiology, Faculty of Pharmacy, Mahidol University, Bangkok 10400, Thailand; ‡ Centre of Biopharmaceutical Science for Healthy Ageing, Faculty of Pharmacy, Mahidol University, Bangkok 10400, Thailand; § Faculty of Pharmacy, Mahidol University, Bangkok 10400, Thailand; ∥ Department of Physiology, Faculty of Medicine Siriraj Hospital, Mahidol University, Bangkok 10400, Thailand; ⊥ Institute of Functional Genomics, CNRS, INSERM, University of Montpellier, 34094 Montpellier, France

**Keywords:** resveratrol, cognition, memory, ROS, inflammation

## Abstract

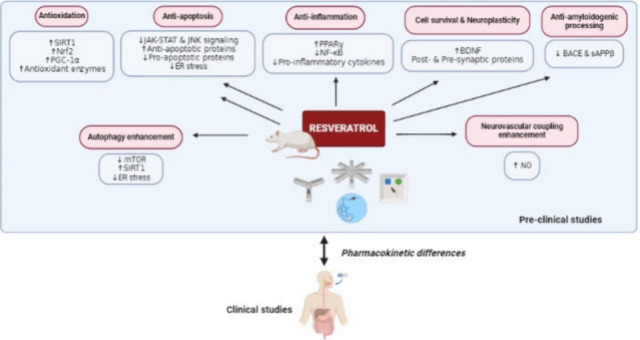

Cognition is essential for daily activities and progressively deteriorates with age due to various factors leading to cognitive decline. This decline often begins with memory impairment and advances to broader cognitive dysfunctions. Resveratrol (RES), a natural phenolic compound found in red wine, has garnered significant attention for its potential to prevent cognitive decline. This review aims to synthesize the latest preclinical data on the cognitive restorative effects of RES. We highlight RES activities from cellular mechanisms to behavioral outcomes. Evidence from various cognitive impairment models demonstrates that RES exerts neuroprotective effects through multiple mechanisms, including anti-inflammatory, antioxidative, anti-apoptotic, and neurotrophic actions, all of which contribute to cognitive enhancement in behavioral studies. Despite the established role of RES in mitigating memory decline, our review identifies a critical gap in behavioral studies regarding cognitive flexibility. Further research in this domain is recommended. Additionally, species-specific pharmacokinetic differences may account for the inconsistencies between preclinical and clinical outcomes, particularly in rats and humans. We propose that formulations designed to delay gut metabolism through enterohepatic circulation could enhance the translational potential of RES. Furthermore, long-term studies are needed to determine the optimal dose capable of maximizing health benefits without raising toxicity during chronic use.

Cognition is a set of mental processes that allows humans to live and thrive in the environment. As social beings, humans rely on cognitive functions such as attention, learning, memory, language, and executive functions to interact each others and comprehend their environment.^1^ When cognitive abilities are impaired, individuals may experience social isolation and a diminished quality of life. Cognitive function declines noticeably with advancing age. The United Nations (UN) predicts that the global population aged 65 and older will exceed 1.5 billion by 2050, indicating a significant rise in the prevalence of age-related cognitive impairment.^2^

In addition to aging, cognitive impairment can be triggered by various factors, including neurodegenerative diseases, cerebral ischemia, head trauma, exposure to agents (such as alcohol, drugs, and heavy metals), stress hormones, and genetic mutations.^3^ A literature review reveals that common contributors to cognitive impairment include neuroinflammation and oxidative stress, which can lead to synapse loss and neuronal cell death.^4,5^ Dysfunctional neuronal circuits impair synaptic transmission and neural connectivity, exacerbating disease pathogenesis. Given these insights, many therapeutic strategies target neuroinflammation and oxidative stress to mitigate cognitive decline. Substances that can prevent or alleviate neurotoxicity, whether derived from natural sources like plants or chemically synthesized, hold the potential for restoring cognitive function or delaying disease progression.

Resveratrol (RES) (3,5,4′-trihydroxystilbene or 5-(2-(4-hydroxyphenyl)vinyl)benzene-1,3-diol) is a naturally occurring phenolic compound predominantly found in red wine, grapes, berries, and beans. Its chemical structure comprises two aromatic rings connected by a methylene bridge, with three phenolic hydroxyl groups attached. RES exists in two isomeric forms, *cis*- and *trans*-RES, with the trans isoform being the most prevalent and exhibiting superior therapeutic potential, particularly in its antioxidant activity.^6^ Exposure to ultraviolet light can convert the trans isoform to the cis isoform, rendering RES light-sensitive and unstable (Figure 1).^7^ This instability presents a challenge for its therapeutic application, necessitating careful handling and formulation to maintain its bioactivity.

**Figure 1:**
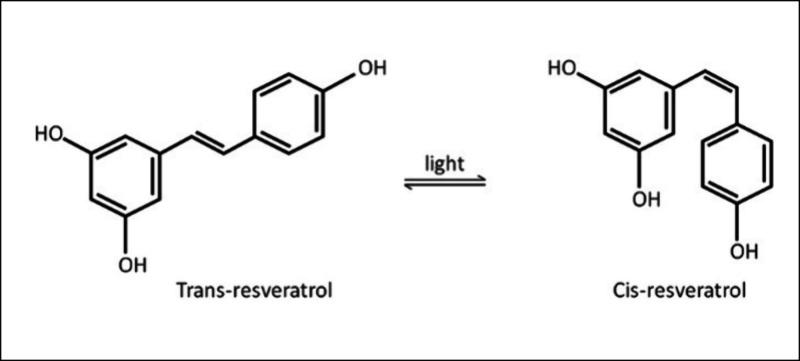
**Resveratrol structure.** RES consists of two isomeric forms that are *cis*-RES and *trans*-RES. The trans isoform is more sensitive to light exposure and rapidly converts to the cis isoform.

Extensive research has investigated RES for its neuroprotective properties, particularly its potential to prevent or mitigate cognitive impairment associated with various neurological disorders. These include Alzheimer’s disease (AD), Parkinson’s disease (PD), epilepsy, and cerebral ischemia. The diverse therapeutic effects of RES are attributed to its ability to modulate multiple biochemical pathways, including those involved in inflammation, oxidative stress, and apoptotic cell death, which are critical in the pathogenesis of these neurodegenerative conditions.^8−11^

The neuroprotective effects of RES against cognitive decline manifest predominantly through its antioxidative properties, which are mediated via the activation of antioxidant enzyme production through the sirtuin 1 (SIRT1) pathway. SIRT1, a member of the nicotinamide adenine dinucleotide (NAD^+^)-dependent histone deacetylase family, plays a critical role in the physiology and pathology of aging and age-related diseases.^12−14^ Within the nervous system, SIRT1 is linked to synaptic plasticity and stress responses, and its levels decrease with advancing age.^15−17^

The antioxidative activity of RES operates through two main mechanisms: enhancing the activity of antioxidant enzymes and acting as a free radical scavenger. Research has shown that RES, by activating SIRT1, increases the levels of key antioxidant enzymes such as superoxide dismutase (SOD), glutathione peroxidase (GPx), glutathione S-transferase (GSTs), and glutathione reductase (GR), while reducing levels of malondialdehyde (MDA), a marker of oxidative stress.^18,19^ Additionally, appropriate doses of RES may activate SIRT1, promoting neurogenesis in the hippocampus.^20,21^

Furthermore, RES influences amyloid precursor protein processing by suppressing amyloid-beta (Aβ) formation, thereby improving memory performance.^22,23^ RES also enhances mitochondrial activity and biogenesis through the SIRT1/AMP-activated protein kinase/Peroxisome proliferator-activated receptor γ coactivator 1-α (SIRT1/AMPK/PGC1-α) pathway and the activation of vitagenes, helping to maintain cellular homeostasis under oxidative stress conditions.^6,24,25^ Additionally, RES’s neuroprotective effects are attributed to the modulation of the Janus kinase/extracellular signal-regulated kinase/signal transducers and activators of transcription (JAK/ERK/STAT) signaling pathway and the cyclic AMP (cAMP)/AMPK/SIRT1 regulatory axis.^26^

In recent years, RES has gained significant attention as a natural compound with potential therapeutic effects for cognitive impairment. This review aims to compile and categorize the latest preclinical research on RES’s ability to mitigate cognitive impairment, from cellular mechanisms to behavioral outcomes. The cognitive assessments focus on rodent models, particularly mice and rats, which exhibit various relevant behaviors, for example, learning behavior. We anticipate that our findings will advance research into RES-based drug discovery.

## 1 The Underlying Mechanisms of Resveratrol in Cognitive Restoration in Cognitive Impairment Models

**Figure 2:**
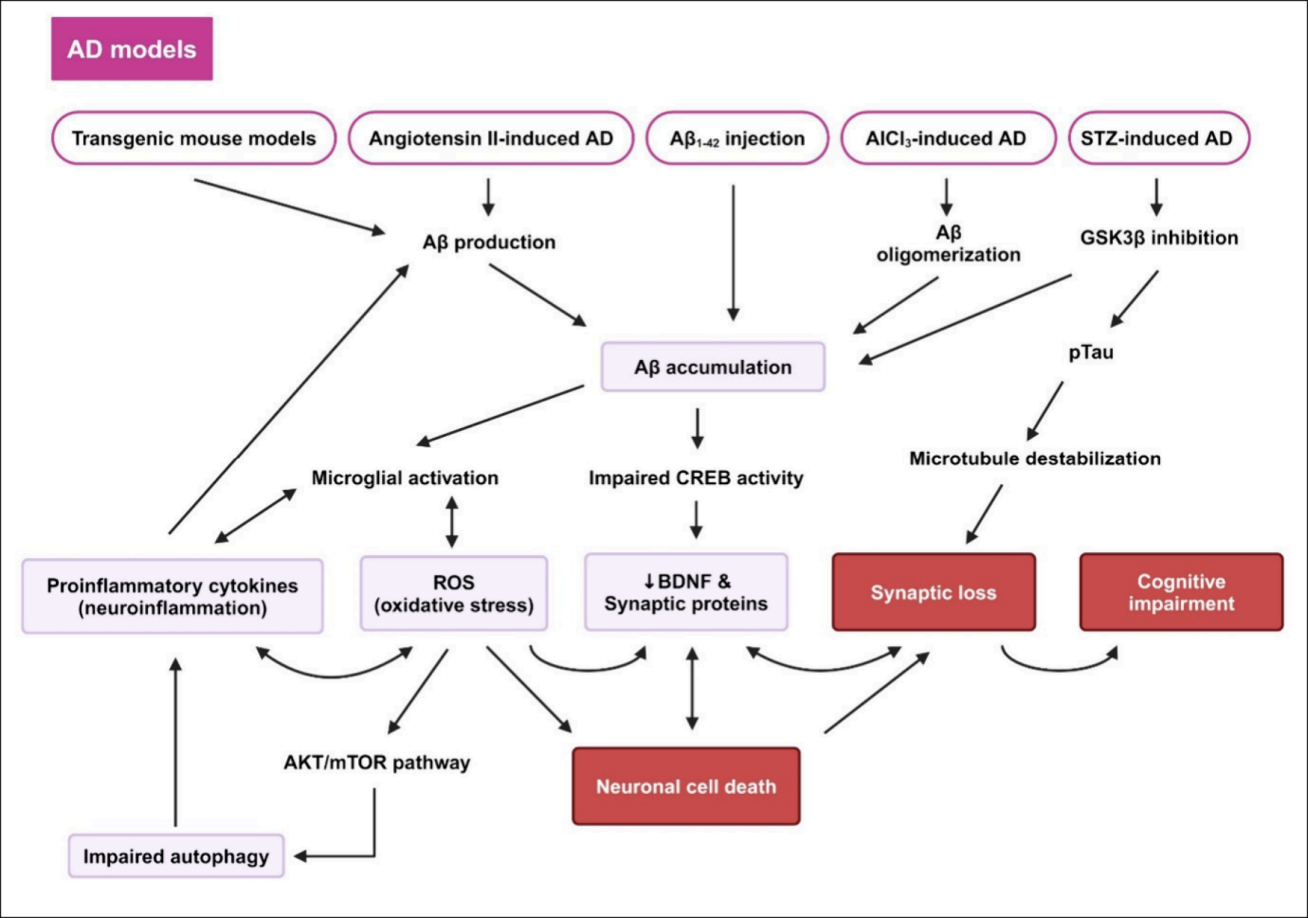
**Mechanisms underlying cognitive impairment in AD models.** Cognitive decline in AD is associated with several mechanisms. The accumulation of misfolded proteins (Aβ and tau) results in significant neuroinflammation and oxidative damage. These pathological events lead to neulonal cell death, synaptic loss, and cognitive impairment. Abbreviations: Aβ_1–42_, amyloid beta peptide fragment 1–42; AD, Alzheimer’s disease; AKT, protein kinase B; AlCl_3_, aluminum chloride; BDNF, brain-derived neurotrophic factor; CREB, cyclic AMP response-binding protein; mTOR, mammalian target of rapamycin; pTau, phosphorylated Tau; ROS, reactive oxygen species; STZ, streptozotocin. Created with BioRender/Mahidol University.

**Figure 3:**
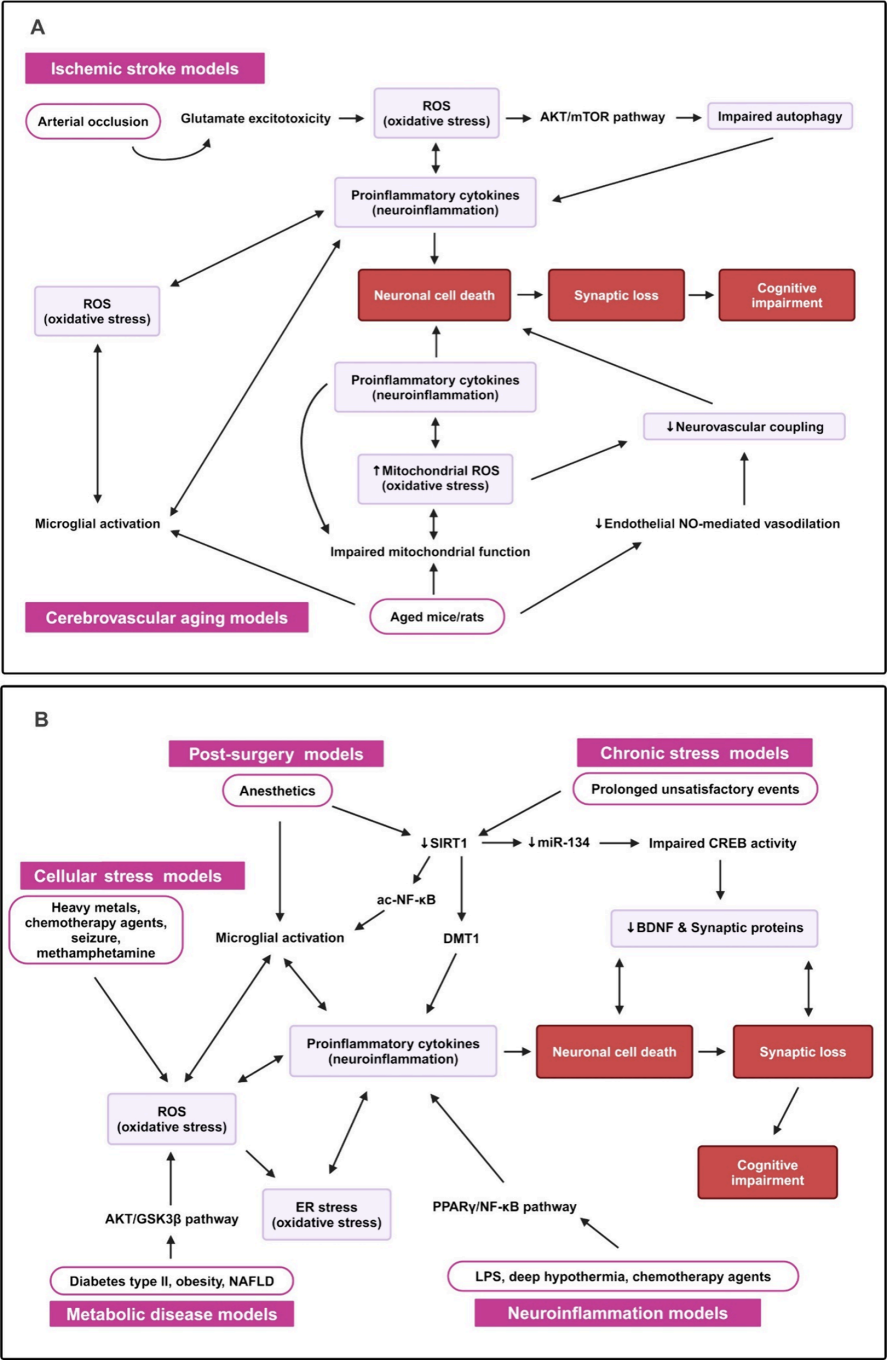
**Mechanisms underlying cognitive impairment in various models.** (A) Ischemic stroke and cerebrovascular aging models. (B) Cellular stress, postsurgery, chronic stress, metabolic disease, and neuroinflammation models. All models share similar detrimental mechanisms, including oxidative stress, neuroinflammation, and impaired autophagy. These pathogenesis events mediate the downregulation of BDNF synthesis, which is crucial for synaptic plasticity and neuronal survival. Reduced BDNF level mediates synaptic depression and neuronal cell death, further exacerbating cognitive impairment. Abbreviations: ac-NF-κB, acetylated nuclear factor kappa B; AKT, protein kinase B; BDNF, brain-derived neurotrophic factor; CREB, cyclic AMP response-binding protein; DMT1, DNA methyltransferase 1; ER, endoplasmic reticulum; LPS, lipopolysaccharide; mTOR, mammalian target of rapamycin; miR-134, microRNA-134; NAFLD, nonalcoholic fatty liver disease; NF-κB, nuclear factor kappa B; NO, nitric oxide; PPARγ, peroxisome proliferator-activated receptor γ; ROS, reactive oxygen species. Created with BioRender/Mahidol University.

The process of normal aging itself can lead to cognitive decline. Moreover, cognitive decline can be precipitated by a multitude of factors, including cerebral vascular dementia, chronic cerebral hypoperfusion, chronic stress, some medications, alcohol, toxins, heavy metals, and surgical operations (Figures 2 and 3). These factors are associated with several events such as mitochondrial dysfunction, endoplasmic reticulum (ER) stress, glial cell activation, and endothelial dysfunction.

All biological changes, whether stemming from aging, neurodegenerative diseases, environmental toxins, or stress, primarily result in neuronal cell death, impaired synaptic transmission, and cognitive dysfunction. Understanding these underlying mechanisms is critical for developing therapeutic strategies to combat cognitive decline. Therefore, RES has been investigated for its potential to restore cognitive function in various models, as developed below.

### 1.1 Cerebrovascular Aging Model

As people age, their brain vasculature undergoes significant physiological and structural changes. These alterations lead to reduced cerebral blood flow, causing neurovascular coupling dyshomeostasis and contributing to cognitive impairment.^27^ For optimal cognitive function, transporting nutrients and oxygen to the brain must align with neuronal demand to maintain homeostasis.

Several studies have demonstrated that exposure to RES can restore neurovascular coupling and vascular endothelial function. RES achieves this by inhibiting the scavenging of nitric oxide (NO), a crucial vasodilator generated by the endothelial cells lining blood vessels.^28−30^ NO plays a vital role in inducing arteriolar dilation, thereby increasing cerebral blood flow and supporting neuronal function.^31^ One study found that administering RES at a dose of 2 mg/kg/day for 4 days to aged mice significantly improved endothelial function and neurovascular coupling. This improvement was mediated by enhanced NO-induced vasodilation, highlighting RES’s potential to counteract age-related vascular dysfunction.^30^ Such findings underscore the therapeutic potential of RES in preserving cognitive function by maintaining vascular health in the aging brain.

The increase in ROS also drives endothelial dysfunction by reducing endothelial nitric oxide synthetase (eNOS) synthesis and enhancing superoxide-NO interactions, which subsequently lead to decreased endothelial NO levels and impaired neurovascular coupling.^32−34^ As individuals age, mitochondrial function declines, resulting in elevated ROS levels and increased inflammatory cytokines. The activation of SIRT1 by RES can potentially mitigate these issues by enhancing the electron transport chain in mitochondria.^33,35,36^

Further evidence from *in vitro* studies demonstrates that RES can activate nuclear factor erythroid 2-related factor 2 (Nrf2) in vascular cells, increase levels of antioxidant enzymes, and promote glutathione (GSH) synthesis.^37,38^ Activation of the Nrf2-dependent pathway is likely a significant contributor to the cerebrovascular protective effects of RES-based treatments. This pathway activation is associated with decreased expression of several genes related to neuroinflammatory pathways, including nuclear factor kappa B (NF-κB), tumor necrosis factor receptor 2 (TNFR2), interleukin-6 (IL-6), NO signaling, and activated microglial markers.^39^

As a result, alleviating these neurotoxic molecules improves endothelial function and restores the balance of neurovascular coupling, potentially slowing the progression of cognitive deterioration in older individuals. These findings underscore the multifaceted role of RES in combating age-related cognitive decline through its antioxidant, anti-inflammatory, and mitochondrial-enhancing properties.

### 1.2 Alzheimer’s Disease Models

The primary symptom of AD is cognitive impairment, which is evident in the early stages of the disease. The pathophysiology of AD is linked to several hypotheses, including the amyloid cascade hypothesis, the tau hypothesis, the inflammatory hypothesis, and the cholinergic and oxidative stress hypotheses. Both the amyloid and tau hypotheses contribute to neuroinflammation and oxidative stress, facilitating hippocampal and cholinergic neuronal cell death, leading to AD pathogenesis.^40^ Conversely, inflammation and oxidative stress can promote the production of amyloid precursor protein^41^ and tau.^42^

AD models are either created genetic manipulation of the mouse genome, such as Tg6799 and 5XFAD mice, or by inducing AD with specific substances like angiotensin-II, aluminum chloride (AlCl_3_), D-galactose, and Aβ_1–42_.^43−45^ Research has shown that the neuroprotective effects of RES in AD stem from its ability to alter APP and tau protein processing pathways and enhance the degradation of misfolded proteins. RES achieves this by decreasing chymotrypsin-like and caspase-like proteasome activity, which improves cognitive function.^43,46,47^ Furthermore, RES has been observed to modulate the oxidative stress and inflammatory responses that are central to AD pathogenesis. By reducing the oxidative damage and inflammatory cytokines, RES helps to protect neuronal cells and maintain synaptic function, which is crucial for cognitive health.

Furthermore, RES has been shown to increase the expression of cyclic AMP response element-binding protein (CREB) mRNA, phosphorylated CREB, and BDNF, which are crucial for neurogenesis and synaptic plasticity. This effect is evidenced by the increased presence of *N*-methyl-d-aspatate receptor (NMDAR)-associated proteins such as synaptic RAS GTPase activating protein (SynGAP) and postsynaptic density 95 (PSD95), both markers of synaptogenesis, in AD mice and rats following RES treatment.^44,45,48^

The administration of RES reduces ROS and proinflammatory cytokines, which may alter APP processing and attenuate β-site APP cleaving enzyme 1 (BACE1) activity, thereby decreasing the production of Aβ_1–42_.^49^ The reduction in amyloid plaque burden subsequently alleviates the activity of kinases such as glycogen synthase kinase-3 beta (GSK3β), cyclin-dependent kinase 5 (CDK-5), and extracellular signal-regulated kinase,^50^ which in turn reduces the expression of phosphorylated tau protein (P-Tau).^48,49,51^ These changes contribute to the preservation of neuronal integrity and cognitive function, highlighting the potential of RES as a therapeutic agent in mitigating AD progression.

By modulating key pathways involved in neuroinflammation, oxidative stress, and synaptic plasticity, RES offers multiple approaches to addressing the complex pathophysiology of AD. Its ability to influence both amyloid and tau pathology, alongside promoting neurogenesis and synaptic health, positions RES as a promising candidate for further exploration in AD treatment strategies.

However, inconsistencies in SIRT1 protein expression were reported. Indeed, SIRT1 levels were not altered in AD mutant mice, whereas^47^ whereas another study reported increased SIRT1 levels following administration of Aβ_1–42_ directly to the hippocampus.^44^ These discrepancies are likely due to differences in the AD model, doses, and routes of administration.

Several factors may account for vatiability in results. First, the pathological induction times differ between the two models. In Wang et al.’s study, Aβ_1–42_ was injected only once, whereas the mutant animals in Chen et al.’s study continuously produced Aβ_1–42_ over time. This continuous production may lead to a chronic state that impacts SIRT1 expression differently than an acute injection. Second, the route of administration plays a critical role. In Wang et al.’s study, RES was administered directly to the hippocampus, bypassing systemic metabolism.^44^ In contrast, Chen et al. administered RES intraperitoneally (IP), which subjects the compound to rapid hepatic metabolism and subsequent excretion of its metabolites.^47^ The intrahippocampal route ensures higher local concentrations of bioactive RES, whereas IP administration likely results in lower bioactive concentrations due to first-pass metabolism.^52^

This difference in bioavailability can significantly affect the outcomes of the studies. Additionally, RES has inherently limited bioavailability, as indicated by its pharmacokinetic profiles.^53^ The concentration of bioactive RES circulating throughout the body after IP administration may be significantly lower than the concentration achieved through direct hippocampal injection. Therefore, the onset and extent of RES’s bioactive effects could vary markedly between the two administration routes, contributing to the observed differences in SIRT1 expression and related outcomes.

### 1.3 Ischemic Stroke Models

Vascular dementia is the second most common cause of dementia, with stroke and transient ischemic attack significantly increasing the incidence of this disease. The resultant diminished cerebral blood flow leads to glutamate-induced excitotoxicity, oxidative stress, and neuroinflammation, which contribute to neuronal cell death and synaptic loss. RES treatment has been shown to restore cognitive function in models of bilateral common carotid artery occlusion and middle cerebral artery occlusion by modulating multiple pathways, including the JAK/ERK/STAT and PI3K/AKT/mTOR pathways.

RES exerts its neuroprotective effects through several mechanisms. It promotes anti-apoptosis by increasing levels of the anti-apoptotic protein B-cell lymphoma 2 (Bcl-2), decreasing pro-apoptotic protein Bcl-2-associated X (Bax) levels, and reducing apoptosis-associated enzyme activity through caspase-3. Furthermore, RES enhances anti-inflammatory responses by lowering levels of pro-inflammatory cytokines such as tumor necrosis factor alpha (TNFα) and IL-6. Additionally, RES promotes antioxidation by reducing MDA levels and increasing SOD levels. Notably, RES also induces autophagy by mediating the degradation of dysfunctional cellular components.^54−56^

These findings reveal that RES’s anti-inflammatory and anti-apoptotic activities are achieved through various mechanisms, including the suppression of the JAK/ERK/STAT and PI3K/AKT/mTOR pathways. The AKT/mTOR signaling pathway plays a crucial role in cell survival, cell cycle regulation, cell proliferation, and apoptosis.^57^ Regulation of the AKT/mTOR signaling pathway has been shown to inhibit or improve the progression of neurodegenerative diseases.^57−59^ In T-cell acute lymphoblastic leukemia (T-ALL) cells, RES inhibits the AKT/mTOR/4E-BP1 pathway and activates p38 and mitogen-activated protein kinase (MAPK) signals, inducing apoptosis.^60^ Similar autophagy activity induced by RES was observed in HeLa cells when mTOR activity was inhibited.^61^

In addition to regulating cell and tissue growth rates, mTOR signaling is essential in inhibiting autophagy and lysosome formation.^62^ Thus, RES’s modulation of autophagy through inhibition of the AKT/mTOR signaling pathway generates a protective effect in the brain. The neuroprotective effects of RES on brain ischemia are evidenced by the restoration of hippocampal long-term potentiation (LTP), a fundamental biological mechanism of hippocampal synaptic plasticity and memory formation. Furthermore, RES improves cholinergic neuron survival and decreases infarct core size, demonstrating its ability to protect the brain from ischemic damage.^63^

Altogether, RES’s multidimensional approach to modulating apoptosis, inflammation, oxidative stress, and autophagy underscores its potential as a therapeutic agent for vascular dementia. The compound’s ability to influence multiple signaling pathways highlights its comprehensive neuroprotective profile, offering hope for mitigating the effects of cerebrovascular insults on cognitive health.

### 1.4 Chronic Stress-Induced Cognitive Impairment Models

Chronic stress is characterized by prolonged exposure to unsatisfactory events, which activates the hypothalamic-pituitary-adrenal (HPA) axis, producing glucocorticoids (GCs). The hippocampus is particularly vulnerable to chronic stress due to its high expression of glucocorticoid receptors. Research has shown that high doses of GCs can significantly alter hippocampal function.^64−66^ In chronic stress-induced cognitive impairment models, animals are subjected to various stressors such as food and water deprivation, immobilization in small cages, and overnight illumination. These ongoing stressors result in elevated GCs hormone production, decreased BDNF levels, and depletion of numerous neurotransmitters, including glutamate and monoamines, linked to cognitive decline and neuropsychiatric disorders.^67,68^

Studies have demonstrated that RES administered intraperitoneally for 3–4 weeks enhances cognitive function in these models.^69,70^ The neuroprotective effects of RES are primarily attributed to the elevation of hippocampal BDNF expression.^69,70^ BDNF is a crucial neurotrophic growth factor in various brain regions, including the hippocampus and cortex. It plays a vital role in neurite outgrowth, cell differentiation, neuronal survival, and synaptic plasticity.^71^

The neuroprotective mechanisms of RES involve stimulating SIRT1/miR-134 and CREB/BDNF signaling pathways in the hippocampus. Activation of SIRT1 leads to the downregulation of miR-134, an epigenetic factor involved in regulating synaptic plasticity, learning, and memory.^69^ Additionally, SIRT1 activation increases CREB and BDNF expression.^69,70^ The SIRT1 pathway, a member of the NAD^+^-dependent deacetylase family, plays a critical role in cellular stress responses, metabolism, and longevity. By activating SIRT1, RES promotes the transcription of genes involved in neuronal survival and plasticity. Increased CREB activity further enhances BDNF transcription, facilitating synaptic plasticity and cognitive function. The upregulation of BDNF supports neuronal health by promoting synaptic growth and resilience against stress-induced damage. This multifaceted neuroprotective action of RES underscores its potential in mitigating chronic stress-induced cognitive impairment through the modulation of critical molecular pathways.

Overall, RES’s ability to enhance hippocampal BDNF expression and activate SIRT1/miR-134 and CREB/BDNF pathways underscores its neuroprotective potential in chronic stress models. These findings highlight the therapeutic promise of RES in preserving cognitive function and combating neuropsychiatric disorders associated with chronic stress.

### 1.5 Neurotoxicity Models

#### 1.5.1 Neuroinflammation

Neuroinflammation is a hallmark feature in the etiology of many neurodegenerative diseases. It is triggered by the activation of many types of glial cells, leading to increased cytokines and chemokines, activation of inflammatory enzymes, cell dysfunction, and apoptosis.^72^ Numerous *in vivo* studies have demonstrated various ways to induce neuroinflammation, such as administering lipopolysaccharide (LPS), chemotherapeutic agents, heavy metals, and deep hypothermia.

LPS, a common endotoxin derived from the outer membrane of Gram-negative bacteria, is frequently used to model neuroinflammation and cognitive impairment. It has been found that RES can restore cognitive impairment induced by LPS. In these studies, the administration of RES reduced the activation of glial cells and elevated the levels of SIRT1 and synaptophysin proteins, which are crucial for synaptic plasticity and cognitive function.^73^ Similarly, RES administration attenuated microglial activation and improved memory performance in a rat model subjected to deep hypothermia, highlighting its antioxidant, anti-inflammatory, and anti-apoptotic properties.^74^

Moreover, RES has been shown to alleviate cognitive deficits induced by chemotherapeutic agents. This improvement is associated with a decrease in pro-inflammatory cytokines and an increase in anti-inflammatory cytokines through the peroxisome proliferator-activated receptor γ (PPARγ) and NF-κB signaling pathway.^75^ This modulation leads to the suppression of pro-inflammatory cytokines and the upregulation of neuroplasticity-related proteins such as BDNF, tyrosine kinase receptor B (TrkB), calmodulin-dependent protein kinase II (CaMKII), and NMDAR subunit 1.^75^ The alteration in the expression of these proteins is closely associated with hippocampal LTP, which is mediated through the activation of NMDAR signaling cascades.^76^ The enhancement of hippocampal LTP, as observed in these studies, correlates with improved cognitive behavior and performance, underscoring the therapeutic potential of RES in mitigating neuroinflammation and restoring cognitive function.

Breifly, the multifaceted neuroprotective effects of RES are evident in several models of neuroinflammation-induced cognitive impairment. By modulating key signaling pathways and reducing the inflammatory response, RES demonstrates significant potential in preserving cognitive function and combating the progression of neurodegenerative diseases. The observed benefits of RES in these studies highlight its role in enhancing synaptic plasticity, promoting neuronal survival, and restoring cognitive performance, thus offering a promising therapeutic strategy for neuroinflammatory conditions.

#### 1.5.2 Cellular Stress

Cellular stress occurs when cells need to alter their biochemistry in response to various stimuli, eventually leading to programmed cell death if not mitigated. Cellular stress can be induced by oxidative, chemical, mechanical, inflammation, hypoxia, endoplasmic reticular stress, or protein misfolding.^77^ In animal experiments, RES has been shown to improve cognitive performance in cellular stress models induced by amphetamine, heavy metal lead (Pb), ROS, paclitaxel, or pentylenetetrazol (PTZ).^78−81^ The increase in SIRT1 protein, Kelch-like ECH-associated protein 1 (Keap1)-Nrf2 signaling pathway activity, and antioxidant enzyme levels demonstrates RES’s antioxidant and anti-apoptotic neuroprotective activities.^78,79^

The Nrf2/Keap1 pathway is crucial in responding to endogenous and exogenous stresses by mediating cytoprotective responses, including the transcription of antioxidant and detoxification enzyme genes.^82,83^ Under normal conditions, Nrf2 is bound to its inhibitory protein, Keap1. However, when inducers like endogenous free radicals (e.g., hydrogen peroxide, NO, and MDA) and extracellular inducers (e.g., heat and UV) modify the cysteine residues of Keap1, it dissociates from Nrf2.^83^ Nrf2 then acts as a transcription factor, binding with Maf in a region of the antioxidant response element (ARE) in the nucleus, which triggers the transcription of phase 2 antioxidant enzyme genes such as heme oxygenase-1 (HO-1), glutamyl-cysteine synthetase-γ (GCSc-γ), γ-glutamylcysteine ligase (γ-GCL), SOD, and NAD(P)H: quinone oxidoreductase 1 (NQO1).^84^

Computational models and molecular docking methods have shown that RES inhibits Keap1’s effects on Nrf2, encouraging Nrf2 to dissociate from Keap1.^79^ The increase of HO-1 and GCSc-γ in the hippocampal tissue of mice serves as evidence for these phenomena.^79,85^ Consequently, phosphorylation of TrkB, AMPK, and PGC-1α, along with increased BDNF protein expression, were observed, contributing to memory recovery.^79^

The AMPK/SIRT1/PGC-1α signaling pathway is proposed as another mechanism through which RES exerts its antioxidant, anti-inflammatory, anti-apoptotic activities, and autophagy. Mitochondrial biogenesis, a process where mitochondria renew themselves and improve functionality, thereby preventing detrimental cascades caused by mitochondrial dysfunction, is a key aspect of this pathway.^86^ PGC-1α, a crucial regulatory protein, can be activated by SIRT1-induced deacetylation and AMPK-induced phosphorylation, initiating mitochondrial biogenesis.^86,87^ Following PGC-1α activation, numerous genes involved in mitochondrial biogenesis, anti-inflammation, anti-apoptosis, anti-amyloidogenic processing, and cell survival/plasticity are transcribed.^88−93^

In cellular stress models, RES increases the expression of SIRT1, phosphorylated SIRT1 (pSIRT1), and PGC-1α. The increased pSIRT1 levels inversely correlate with MDA levels, indicating a reduction in oxidative stress.^94,95^ RES also directly activates AMPK, which controls antioxidant responses by regulating mitochondrial regulators like PGC-1α and SIRT1.^90,94,95^ In a study on mice with Pb-induced cognitive impairment, RES treatment restored SIRT1 and AMPK phosphorylation levels, demonstrating its ability to activate the SIRT1/AMPK pathway.^78^

In the Pb-induced amyloid-beta 1–40 (Aβ_1–40_) formation model, RES inhibited BACE expression, a key enzyme in the amyloidogenic pathway that facilitates AD pathogenesis. This inhibition led to a decline in Aβ_1–40_ levels in the rat brain.^78^ Additionally, RES increased glutathione (GSH) levels and decreased superoxide radical (O_2_^•–^) production in models exposed to PTZ, arsenic (As), and manganese,^96^ supporting its antioxidant activity.^80,97,98^ However, in the PTZ-induced model, the expression of BDNF, CREB, and c-Fos proteins did not increase, contrary to findings by Lang et al. (2022), where BDNF protein was enhanced following RES treatment in Mn-exposed mice.^99^

Overall, the neuroprotective effects of RES in cellular stress models are multifaceted, involving antioxidant, anti-inflammatory, and anti-apoptotic activities and enhanced mitochondrial function. By modulating key signaling pathways and reducing oxidative stress and inflammation, RES demonstrates significant potential in preserving cognitive function and mitigating the progression of neurodegenerative diseases.

### 1.6 Postoperative Cognitive Dysfunction Models

Postoperative cognitive dysfunction (POCD) refers to a decline in cognitive function that can occur after surgery, predominantly affecting older adults. Although the precise mechanism underlying POCD remains unclear, several hypotheses suggest potential contributing factors, including neuroinflammation, oxidative stress, substance neurotoxicity, vascular dysfunction, neurovascular coupling, and blood-brain barrier disruption.^100^

Anesthetics such as pentobarbital, sevoflurane, and isoflurane are commonly used in POCD models to induce cognitive impairment. Research has demonstrated that RES can improve cognitive function in POCD rat models.^101,102^ This neuroprotective effect of RES is attributed to several mechanisms, including the upregulation of SIRT1, suppression of pro-inflammatory cytokines (IL-1β, IL-6, and TNF-α), and inhibition of the ER stress pathway.^103,104^

SIRT1 is a protein crucial in cellular stress resistance, metabolism, and longevity. Upregulation of SIRT1 following RES treatment has been shown to mitigate cognitive decline by various means. SIRT1 enhances the deacetylation of transcription factors, thereby regulating genes involved in stress resistance, inflammation, and neuronal function. In the context of POCD, RES-induced SIRT1 upregulation leads to a decrease in pro-inflammatory cytokines, contributing to antineuroinflammation and cognitive restoration.^102^ RES alleviates inflammation and promotes neuronal health by suppressing the release of IL-1β, IL-6, and TNF-α.

Furthermore, the ER stress pathway is another critical factor in POCD. ER stress occurs when there is an accumulation of misfolded or unfolded proteins in the ER, leading to the activation of intracellular signaling proteins such as inositol-requiring enzyme 1α (IRE1α), protein kinase R-like ER kinase (PERK), C/EBP homologous protein (CHOP), and unspliced X-box binding protein 1 (XBP-1u). These proteins contribute to cellular stress and apoptosis, exacerbating cognitive decline. RES treatment has been shown to inhibit the activity of these proteins, thereby reducing ER stress and its detrimental effects on neurons.^103^ Moreover, RES promotes neuronal maturation and proliferation by facilitating the production of the transcription factor Sox2, which is essential for neurogenesis and the maintenance of stem cells. RES activates SIRT1 by enhancing Sox2 expression, thereby supporting new neuron generation and repairing damaged neural tissues.^104^

The neuroprotective effects of RES in POCD are multifaceted. By upregulating SIRT1, RES reduces neuroinflammation and ER stress and promotes neurogenesis and neuronal repair. These combined actions contribute to preserving and improving cognitive function following surgery. Conclusively, RES shows promising potential in mitigating POCD through its various neuroprotective mechanisms. By targeting critical pathways involved in neuroinflammation, oxidative stress, ER stress, and neurogenesis, RES helps to maintain cognitive function and counteract the adverse effects of surgical procedures on the brain. These findings underscore the therapeutic potential of RES for older adults undergoing surgery, highlighting its importance in addressing cognitive decline associated with POCD.

### 1.7 Metabolic Disease Models

Several reports have demonstrated that neuroendocrine dysfunction caused by metabolic diseases can mediate cognitive impairment.^105−107^ Diabetes, a prevalent metabolic disease, significantly impacts cognitive function when not adequately controlled. Fundamental pathological mechanisms include the formation of advanced glycation end products (AGEs), insulin resistance, hyperglycemia, and hypoglycemia, which collectively mediate inflammation, oxidative stress, and microvascular damage within the brain.^41,108^

Experimental models using rodents have provided insights into the potential therapeutic effects of RES in ameliorating cognitive impairments associated with metabolic diseases. Various treatment durations of RES have been administered in rodent models induced by streptozotocin,^85,109^ fructose feeding,^110^ or a high-fat diet (HFD).^85,90,111^ RES demonstrated significant neuroprotective activities in these models that contributed to improved cognitive functions. This improvement is linked to the upregulation of oxidative stress-related proteins such as SIRT1 and its downstream targets, including PGC-1α and fibronectin type III domain-containing protein 5 (FNDC5).^92,112^

Additionally, RES activated Nrf-2,^86^ which in turn upregulated antioxidant enzymes like HO-1 and NQO-1, as well as metallothionein genes.^97^ RES also enhanced the activities of SOD, catalase (CAT),^113^ glutathione peroxidase (GPx), and NADPH oxidase.^86,112^ Furthermore, RES treatment resulted in the depletion of pro-inflammatory cytokines such as TNFα and interleukin-1 beta (IL-1β), along with oxidative stress markers including MDA, 8-hydroxyguanosine (8-OHdG), and 3-nitrotyrosine (3NT).^84,90,109,111,112,114^ This anti-inflammatory and antioxidative profile underscores RES’s potential to mitigate cognitive decline due to metabolic diseases.

RES also exhibited beneficial effects on ER stress markers. By reducing the levels of phosphorylated eukaryotic initiation factor 2 (p-eIF2)/eIF2, activating transcription factor 4 (ATF4), and CHOP, RES helps restore ER function and promotes autophagy activity.^112^ Additionally, RES altered the expression of microRNA-21 (miR-21), which is crucial for post-transcriptional regulation associated with neurogenesis, cell survival, proliferation, and anti-apoptosis.^113^

Regarding synaptic function, RES modulated the expression of several synaptic proteins, including BDNF, Copine 6, and phosphorylated-catenin/catenin proteins. It also decreased phosphorylated glycogen synthase kinase 3 beta (p-GSK3β)/GSK3β proteins, further enhancing synaptic activity.^85,90,109,111,113^ These changes were associated with increased cell density in the hippocampus and temporal-parietal cortex.^110,113^

Finally, the neuroprotective properties of RES in metabolic disease models highlight its potential therapeutic value in improving cognitive function. By modulating oxidative stress, inflammatory pathways, ER stress, and synaptic activity, RES offers a multifaceted approach to combat cognitive decline associated with metabolic disorders. Further research is essential to fully elucidate these mechanisms and translate these findings into clinical applications.

## 2 Mechanisms of RES Underlying Cognitive Restoration

This review shows that RES exhibits numerous neuroprotective properties, which significantly contribute to its potential as a therapeutic agent in preventing cognitive impairment, as shown in Table 1.

**Table 1:** Effect of Resveratrol on Biochemical Analyses

model of study	dose	duration	biochemical analyses	ref
Cerebrovascular aging model (24 months old male C57BL/6 mice)	2 mg/kg/day	4 days	↑ CBF via NO mediation (restored neurovascular coupling)	(30)
	3,4′,5-trihydroxystilbene (retro orbital)			
Cerebrovascular aging model (6 months old male Sprague-Dawley rats)	1.25 mg/rat/day	5 months	↑ Microglial M2 polarization marker genes; *IL-4*, *IL-10*, *IL-13*, *IL-25*, *CD206*, and *IGF-1*	(39)
	3,5,4′-trihydroxy-*trans*-stilbene (drinking water)		↓ Microglial M1 polarization marker gene; *IL-23*, *TNF-α*, and *IFN-α*	
			↓ Inflammatory pathway: Eicosanoid signaling, MiF-mediated innate immunity, NF-κB signaling, dendritic cell maturation, TNFR2 signaling, and IL6	
			↑ CBF during performing NOR task	
AD model: Hypertension-induced AD pathogenesis by using Angiotensin-II (10 weeks old male Wistar Kyoto rats)	10 mg/kg/day	14 days	↑ BDNF	(43)
	3,5,4′-trihydroxy-*trans*-stilbene (intragastric)		↓ NOX2	
			↓ Superoxide	
			↑ SOD2	
			↓ AT1R, Aβ precursors, and active caspase 3	
			↓ p-Akt, p-GSK-3β, and p-Tau	
AD model: APP/presenilin 1 double transgenic mouse model (18–22 g, 2.5 months old male Tg6799 mice)	60 mg/kg/day Resveratrol (intragastric)	60 days	↓ Amyloid plaques in hippocampus	(47)
			↓ Aβ_42_ and Aβ_40_	
			↓ APP, sAPPα, sAPPβ, and BACE1	
			↔ SIRT1	
AD model: AlCl_3_ and D-galactose-induced sporadic AD mouse model (18–22 g, adult normal male Swiss mice)	40 mg/kg/day 3,4′,5-trihydroxystilbene (intraperitoneal)	10 weeks	↑ BDNF protein	(45)
			↔ CREB protein	
AD model: a high-fat diet triggering AD progression in human APP and PSEN1 transgenic mouse model (5XFAD mouse)	120 mg/kg/day *trans*-resveratrol (intragastric)	16 weeks	↔ APP protein	(46)
			↓ C-terminal fragments of APP protein	
			↓ Fibrillary amyloid plaques	
			↓ BACE1 and sAPPβ proteins	
			↓ p-Tau protein	
			↓ Chymotrypsin-like and Caspase-like proteasome activities*	
			↔ Trypsin-like activities*	
			↓ β5 and β5i subunits of 20S proteasome proteins*	
			↔ β1 and β2 catalytic subunits of 20S proteasome proteins*	
			↓ 20S proteasome protein*	
			*Alteration of proteolytic activity of the ubiquitin-proteasome system	
AD model: Intrahippocampal injection of Aβ_1–42_ (220–240 g, adult male Sprague-Dawley rats)	0.5, 1.25, 5, 22, 44 μmol 3,4,5′-trihydroxy-*trans*-stilbene (intrahippocampal)	Single dose	↑ Hippocampal LTP	(44)
			↑ SIRT1 protein	
			↑ p-CREB protein	
AD model: Intrahippocampal injection of STZ (3 months old male C57BL/6 mice, *n* = 13/group)	25 mg/kg 3,5,4′-trihydroxy-*trans*-stilbene (intragastric)	5 weeks	↓ Aβ_1–42_ protein expression	(48)
			↓ Tau and hyperphosphorylated tau protein expression	
			↑ SynGAP, PSD95, and BDNF protein expression	
			↓ Iba1 microglial activation marker	
Ischemic stroke model: Bilateral common carotid artery occlusion (2 months old male Sprague-Dawley rats)	10 and 20 mg/kg/day 3,5,4′-trihydroxy-*trans*-stilbene (intraperitoneal)	4 weeks	↑ SOD levels	(56)
			↓ MDA levels	
			↑ Bcl-2 protein	
			↓ Cleaved caspase-3 and Bax proteins	
Ischemic stroke model: Chronic cerebral hypoperfusion induced by bilateral common carotid artery occlusion (260–300 g, ∼ 10–18 weeks old male Sprague-Dawley rats)	50 mg/kg/day resveratrol (intragastric)	3, 6, and 9 weeks	↑ SOD and GSH levels	(54)
			↓ MDA levels	
			↑ Bcl-2 protein	
			↓ Cleaved caspase-3 and Bax proteins	
			↓ Akt, mTOR, S6K1, and 4E-BP1 proteins	
			↓ LC3B and Beclin-1 proteins (cellular autophagy markers)	
			*All protein expressions were reversed after administration of PI3K inhibitor	
Ischemic stroke model: Middle cerebral ischemia/reperfusion (260–300 g, ∼ 10–18 weeks old Sprague-Dawley rats)	20 mg/kg/day *trans*-resveratrol (intraperitoneal)	7 days	↑ Hippocampal neuron survival	(55)
			↑ SOD levels	
			↓ MDA levels	
			↓ TNF-α and IL-6 levels	
			↓ Bax protein	
			↑ Bcl-2 protein	
			↓ p-JAK, p-STAT, p-ERK, and p-JNK proteins	
Ischemic stroke: Transient middle cerebral artery occlusion (2–3 months old male Sprague-Dawley rats, *n* = 5–7/group)	50 mg/kg 3,4,5′-trihydroxy-*trans*-stilbene (intragastric)	Single does	↑ hippocampal LTP (in mice)	(63)
			↓ Infact size (in rats)	
			↓ Central cholinergic cell loss (Basal forebrain) (in rats)	
Chronic unpredictable mild stress model (180–200 g, ∼ 6–8 weeks old male Sprague-Dawley rats)	40 and 80 mg/kg/day 3,4′,5-trihydroxy-*trans*-stilbene (intraperitoneal)	4 weeks	↑ BDNF, p-CREB, CREB and SIRT1 proteins	(69)
			↓ miR-134 (associated with CREB/BDNF expression) protein	
Chronic restraint stress-induced cognitive impairment model (8 weeks old male Wistar rats)	80 mg/kg/day resveratrol (intraperitoneal)	21 days	↑ *BDNF* mRNA and protein	(70)
Neuroinflammation model: LPS (8 weeks old male ICR mice)	5, 10, and 20 mg/kg/day	5 days	↓ GFAP (activated astrocyte marker)	(73)
	3,5,4′-trihydroxystilbene (intraperitoneal)		↓ Iba-1 (activated microglial marker)	
			↑ Synaptophysin protein	
			↑ SIRT1 protein	
Neuroinflammation model: deep hypothermia (350–400 g, ∼ 12–14 weeks old male Sprague-Dawley rats)	30 mg/kg/day resveratrol (intravenous)	7 days	↓ IL-1β, IL-6, and TNF-α levels	(74)
			↓ Microglial activation	
			↓ Cell apoptosis	
			↑ A20 protein	
			↓ TRAF6, IRP1, p-IκBα/IκBα, and p-NF-κB/NF-κB p65 proteins	
Neuroinflammation model: chemotherapeutic regimens (18–20 g, ∼ 6–9 weeks old female C57BL/6 mice)	50 and 100 mg/kg/day resveratrol (intragastric)	3 weeks	↓ IL-6, TNF-α, IL-4 and IL-10 levels	(75)
			↑ PPARγ and p65 proteins	
			↓ p-p65 and p-IκBα proteins	
			↓ p-CaMKII protein	
			↑ GABAAR, NMDAR1, CaMKII, BDNF, and TrkB proteins	
Cellular stress model: Heavy metal lead exposure (16 months old male C57BL/6 mice)	50 mg/kg/every other day resveratrol (intragastric)	48 weeks	↑ mature BDNF protein	(78)
			↑ p-TrkB/TrkB ratio	
			↓ BACE1 and Aβ_1–40_ levels	
			↑ SIRT and p-SIRT1 protein	
			↑ p-AMPK/AMPK ratio	
			↑ PGC-1α protein	
			↓ MDA and 8-OHdG levels	
			↑ GSH-Px, CAT and GSH levels	
Cellular stress model: Methamphetamine (18–22 g, ∼ 6–9 weeks old C57BL/6 mice)	10 and 100 mg/kg/day 3,5,4′-trihydroxy-*trans*-stilbene (intragastric)	17 days	↓ MDA levels	(79)
			↑ SOD, GSH and total antioxidant activities	
			↑ Nrf2, HO-1, and GCSc-γ protein	
			↓ Keap1 protein	
			↑ Bcl-2 protein	
			↓ Cleaved caspase-3 and Bax proteins	
Cellular stress model: pentylenetetrazole-induced epileptic seizure (250–400 g, ∼ 9–21 weeks old male Wistar rats)	40 mg/kg/day resveratrol (drinking water)	31 days	↑ GSH levels	(80)
			↓ Superoxide radicals	
			↔ MDA and myeloperoxidase (MPO) levels	
			↓ TNF-α and IL-6 levels	
			↓ CREB, BDNF, and cFos proteins	
			↔ GABA activity	
Cellular stress model: Paclitaxel (chemotherapy agent) (20–24 g, 6–8 weeks old male C57BL/6 mice, *n* = 10/group)	90 mmol/L/day resveratrol (intracerebroventricular)	7 days	↓ RIP3 and MLKL protein expressions (specific molecules of necrotic apoptosis)	(81)
			↓ NOX2 and NOX4 protein expressions (ROS)	
			↑ SIRT1 and PGC-1α protein expressions	
			↑ IL-4 and IL-10 protein levels (↑ Microglial M2 polarization)	
			↑ *BDNF* and *PSD95* mRNA expression	
			↑ PSD95 protein expression	
			↑ Dendritic spines	
Cellular stress model: Manganese exposure (20–25 g, 9–10 weeks old C57BL/6 mice, *n* = 10/group)	30 mg/kg/day	42 days	↑ Nrf2 and HO-1 protein expression	(97)
	3,4′,5-trihydroxystilbene (intragastric)		↓ Intracellular ROS, MDA	
			↑ hippocampal antioxidant enzymes (SOD, CAT, and GSH)	
			↓ GFAP protein expression (activated astrocyte marker)	
			↓ Iba1 protein expression (activated microglial marker)	
			↓ IL-1β, TNF-α, and COX-2 levels	
			↓ p-NF-κB/NF-κB, NF-κB, and ac- NF-κB proteins	
			↓ p-STAT3/STAT3 protein	
			↓ p-JNK/JNK protein	
			↑ SIRT1 protein	
Cellular stress model: Arsenic exposure (200–230 g, adult male Wistar rats, *n* = 8/group)	10 and 20 mg/kg/day	21 days	↑ FRAP activity (test for antioxidant capacity assessment)	(98)
	3,4′,5-trihydroxy-*trans*-stilbene (intraperitoneal)		↑ GSH level	
			↓ MDA level	
Cellular stress model: Manganese exposure (23–25 g, 6 weeks old adult Kunming mice, male:female ratio = 1:1)	30 mg/kg/day	6 weeks	↑ PGC-1α, ULK1, LC3-II proteins (autophagy)	(99)
	3,4′,5-trihydroxystilbene (intragastric)		↓ p62 protein (autophagy)	
			↓ p-NF-κB/NF-κB	
			↑ BDNF protein	
			↓ *iNOS*, *TNF-α*, *IL-6*, and *IL-1β* mRNA expression (↓ Microglial M1 polarization)	
			↑ *TGF-β*, *IL-4*, *IL-10*, and *Arg1* mRNA expression (↑ Microglial M2 polarization)	
			↓ STAT6 activity (inflammatory pathway)	
POCD model (21 months old male Sprague-Dawley rats)	10 mg/kg/day resveratrol (intraperitoneal)	7 days	↓ CD86 (marker of cytotoxic M1 microglia) and SOCS3 (marker of immunomodulatory M2b microglia)	(101)
			↑ CD206 (marker of repair M2a microglia)	
			↑ DNMT1 and SIRT1 proteins	
			↓ ac- NF-κB protein	
			↓ IL-1β, IL-6 and TNF-α levels	
POCD model (21 months old male Sprague-Dawley rats)	10 mg/kg/day	15 days	↑ Neuronal SIRT1 protein	(102)
	3,4,5-trihydroxystilbene (intraperitoneal)		↓ ac-tau (k280), ac-tau (k686), p-tau (AT8) protein	
POCD model (18 months old male C57BL/6 mice)	100 mg/kg/day resveratrol (intraperitoneal)	7 days	↑ SIRT1 protein	(103)
			↓ ER stress-related proteins; IRE1α, PERK, CHOP, and XBP-1u	
			↓ NF-κB protein	
POCD model (18 months old male C57BL/6 mice)	50 mg/kg/day resveratrol (intraperitoneal)	7 days	↑ SIRT1 activity	(104)
			↑ Sox2+GFAP positive cell (astrogenesis)	
			↑ Sox2+BrdU-positive cell (neurogenesis)	
POCD model (16 months old male C57BL/6 mice)	10 mg/kg/day resveratrol (intraperitoneal)	5 days	↑ SIRT1 protein	(115)
			↓ Bax protein	
			↑ Beclin-1 protein (autophagy marker)	
			↑ LC3-II/LC3-I ratio (autophagy marker)	
			↓ P62 protein (a selective autophagic substrate)	
Metabolic disease model: Diabetes mellitus type II induced by STZ and a high-fat diet (8 weeks old male ICR mice)	30 mg/kg/every other day resveratrol (intragastric)	4 months	↑ *Nrf2* mRNA and protein	(85)
			↑ *NQO-1*, *OH-1*, and *MT* mRNA and proteins	
			↑ SOD and CAT activities	
			↓ 8-OHdG, MDA, and 3-NT levels	
			↓ TNF-α and IL-1β proteins	
			↑ Hippocampal neurons and synapses in CA1 area	
Metabolic disease model: Obesity induced by a high-fat diet (15 months old male C57BL/6 mice)	100 mg/kg/day resveratrol (intraperitoneal)	7 days	↑ SIRT1 protein	(90)
			↑ PGC-1α, FNDC5, and BDNF proteins	
Metabolic disease model: Diabetic model induced by STZ (250–300 g, adult male Wistar rats)	20 mg/kg/day resveratrol (intraperitoneal)	4 weeks	↑ Hippocampal *BDNF* mRNA	(109)
			↓ Hippocampal *TNF-α* and *IL-1β* mRNA	
Metabolic disease model: Fructose feeding model (6–12 months old Wistar rats)	2 mg/kg/day natural resveratrol (intragastric)	16 weeks	↑ Temporal-parietal cortex thickness and neuronal density	(110)
Metabolic disease model: NAFLD model induced by a high-fat diet (2 months old male Sprague-Dawley rats)	15 mg/kg/day 3,5,4′-trihydroxy-*trans*-stilbene (intragastric)	4 weeks	↑ Copine 6 protein (a regulator of spontaneous neurotransmission)	(111)
			↑ p-catenin/catenin ratio	
			↓ p-GSK3β/GSK3β ratio	
			↔ Cyclin D1 protein	
Metabolic disease model: Diabetes mellitus type II induced by STZ and a high-fat diet (22–25 g, 8 weeks old, male C57BL/6 mice, *n* = 12/group)	5 mg/kg/day	8 weeks	↓ p-elF2α/elF2α, ATF4, and CHOP protein expression (ER stress)	(112)
	3,4′,5-trihydroxy-*trans*-stilbene (drinking water)		↓ LDH activity (marker of cell damage)	
			↑ Bcl-2 protein expression	
			↓ Bax, caspase-3, p16, p21, and p53 protein expression	
Metabolic disease model: Diabetes mellitus type II induced by streptozotocin and a high-fat diet (125–150 g, adult male Albino Wistar rat, *n* = 10/group)	20 mg/kg/day resveratrol (intraperitoneal)	4 weeks	↓ HOMA-IR (insulin resistance index)	(113)
			↓ Serum glucose	
			↓ Serum and hippocampal AGE levels	
			↓ Hippocampal MDA levels	
			↑ Hippocampal total antioxidant capacity	
			↓ Hippocampal GSK3β	
			↑ Hippocampal miR-21	
			↑ Hippocampal neuron density	
Metabolic disease model: Diabetes mellitus type II induced by STZ and a high-fat diet (180–220 g,12–14 weeks old, adult male Sprague-Dawley rats, *n* = 42/group)	50, 100 mg/kg/day resveratrol (intraperitoneal)	4 weeks	↓ Blood glucose levels	(114)
			↓ HbA1c levels (glycosylated hemoglobin)	
			↓ Total cholesterol, plasma LDL, and plasma TG levels	
			↑ Plasma HDL levels	
			↑ Plasma CAT, SOD, GPx, and GSH levels	
			↓ Plasma MDA levels	
			↓ Nitrite levels and ChE activities in plasma, hippocampus, and cerebral cortex	
			↓ TNF-α and TGF-β1 levels in plasma, hippocampus, and cerebral cortex	
			↑ SIRT1 levels in plasma, hippocampus, and cerebral cortex	

### Antioxidation

RES enhances the brain’s antioxidative capacity by upregulating the expression and activity of key antioxidant enzymes such as SOD, CAT, and GPx. These enzymes are critical in neutralizing ROS and reducing oxidative damage to neuronal cells.^86,92,112,113^ Additionally, RES activates Nrf-2, a master regulator of the antioxidant response, further stimulating the expression of antioxidant genes.^86^

### Anti-apoptosis

RES demonstrates significant anti-apoptotic effects by modulating signaling pathways involved in cell survival and apoptosis. The activation of SIRT1 by RES enhances neuronal survival by deacetylating and activating transcription factors such as FOXO and p53, which are critical for cell survival under stress conditions. RES also reduces the expression of pro-apoptotic proteins, including Bax and caspase 3, via the alteration of ER stress.^103,112,115^

### Anti-inflammation

RES exerts robust anti-inflammatory effects by inhibiting the production and release of pro-inflammatory cytokines, including TNF-α, IL-1β, and IL-6. These cytokines are critical mediators of neuroinflammation, which can lead to neuronal damage and cognitive impairment. By suppressing these inflammatory responses, RES helps to protect neuronal integrity and function.^102,109,111^

### Anti-amyloidogenic Processing

RES decreases the levels of BACE1 and soluble amyloid precursor protein beta (sAPPβ), reducing amyloid plaque formation. This anti-amyloidogenic effect is crucial in preventing the aggregation of Aβ, which are implicated in the pathogenesis of AD.^46,49,78^

Beyond these primary mechanisms, RES also contributes to neuroprotection through several other pathways:

### Cerebral Blood Flow

RES has been shown to improve cerebral blood flow, which is crucial for maintaining adequate oxygen and nutrient supply to the brain. Enhanced blood flow supports neuronal health and function, reducing the risk of cognitive decline.^30,39^

### Autophagy

**Figure 4:**
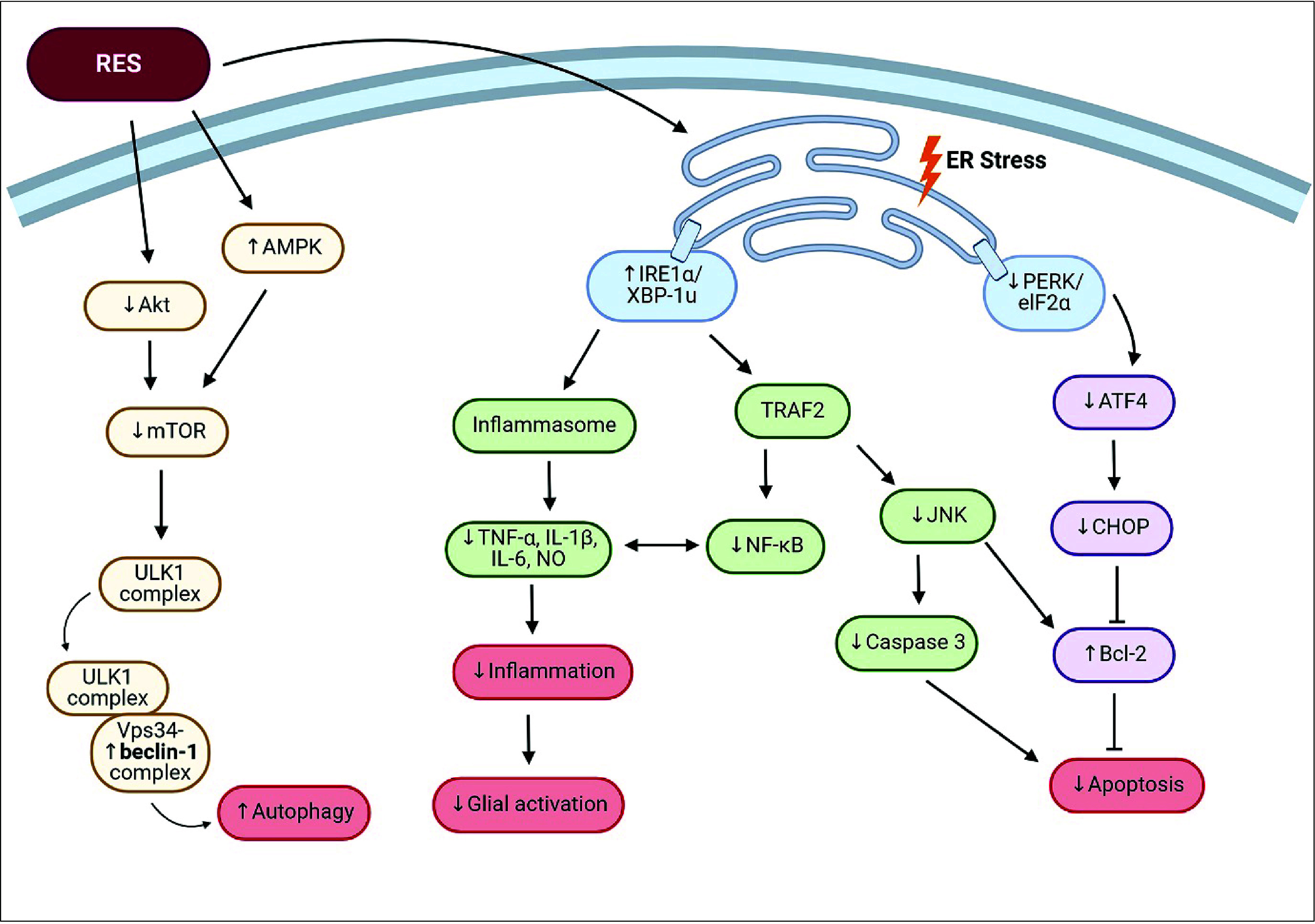
**Mechanisms of resveratrol on ER stress, apoptosis, and autophagy.** ER stress drives downstream signaling cascades involving PERK/eIF2α and IRE1α/XBP-1u. These two signaling cascades’ activation essentially causes neuroinflammation and apoptosis. However, RES can reverse all events by changing the protein expression associated with these downstream signaling cascades. Additionally, RES enhances autophagy, which reduces ER stress by blocking mTOR activity. Abbreviations: Akt, protein kinase B; AMPK, AMP-activated protein kinase; ATF4, activating transcription factor 4; Bcl2, B-cell lymphoma 2; CHOP, C/EBP homologous protein; eIF2α, eukaryotic initiation factor 2 alpha; ER, endoplasmic reticulum; IL-1β, interleukin-1 beta; IL-6, interleukin-6; IRE1α, inositol-requiring enzyme 1 alpha; JNK, c-Jun N-terminal kinase; mTOR, mammalian target of rapamycin; NF-κB, nuclear factor kappa B; PERK, protein kinase R-like endoplasmic reticulum kinase; TNFα, tumor necrosis factor alpha; TRAF2, TNF receptor associated factor 2; ULK1, unc-51-like-kinase 1; Vps34, class 3 PI3 kinase (PI3KC3); XBP-1u; unspliced X-box binding protein 1. Created with BioRender/Mahidol University.

RES promotes autophagy, a cellular process that removes damaged proteins and organelles, by altering the mTOR-autophagy-ER stress signaling pathway (Figure 4). These processes maintain cellular homeostasis and preventing neurodegeneration. By facilitating the clearance of toxic cellular components, RES helps preserve neuronal function and viability.^99,112,116^

### Cell Survival and Neural Plasticity

RES enhances cell survival and neural plasticity by modulating the expression of proteins involved in synaptic function and plasticity. These include BDNF, Copine 6, and phosphorylated catenin/catenin proteins, which are essential for synaptic health and cognitive function. Additionally, RES reduces the activity of GSK3β, further supporting synaptic activity and plasticity.^85,90,109,111,113^

### MicroRNA Regulation

RES influences the expression of microRNAs, such as miR-21 and miR-134, which regulate post-transcriptional processes associated with neurogenesis, cell survival, proliferation, and anti-apoptosis. This regulatory effect contributes to RES’s overall neuroprotective action.^69,113^

**Figure 5:**
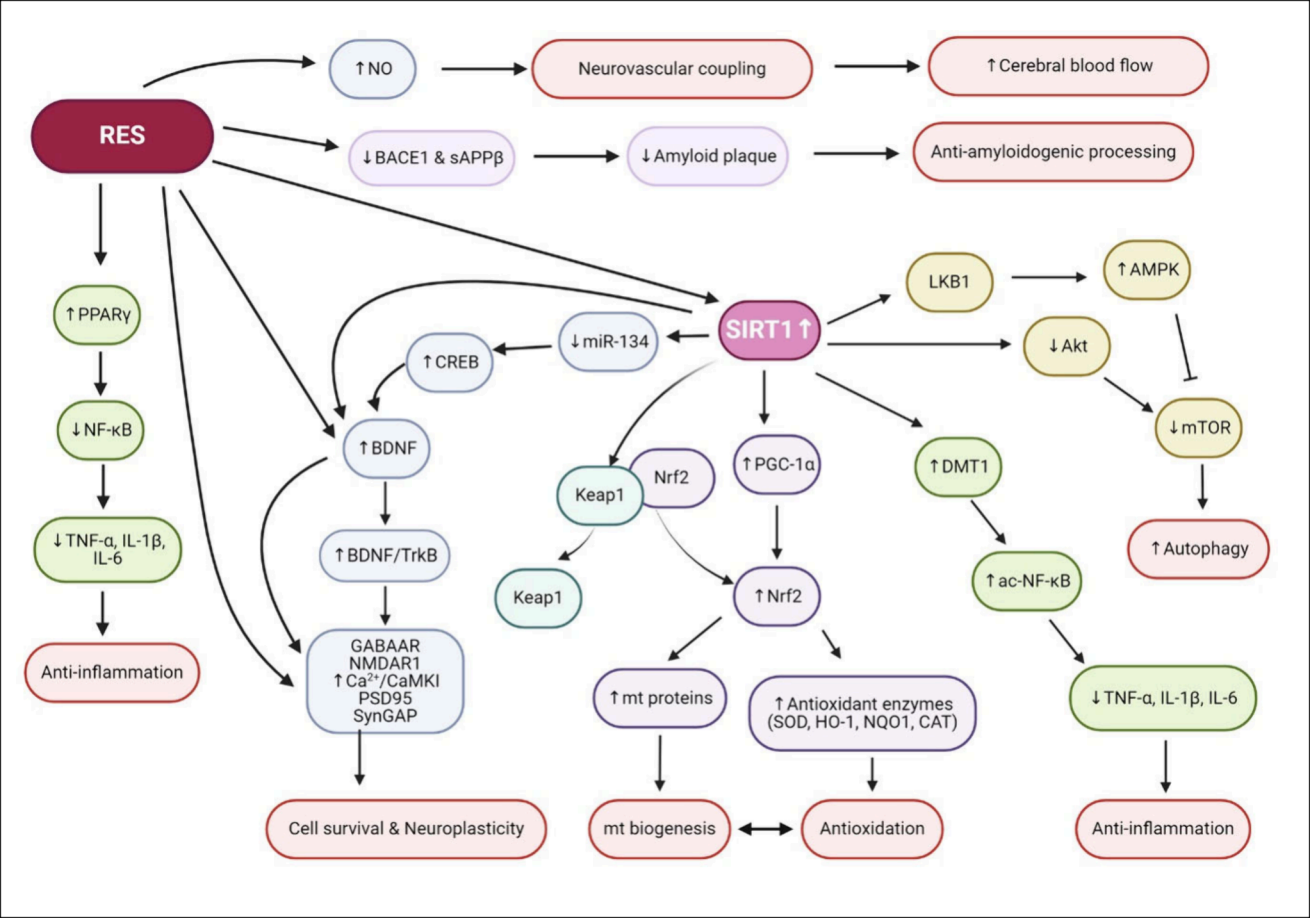
**Mechanisms of resveratrol on cognitive decline restoration.** RES exhibits neuroprotective effects across many pathways. SIRT1 is a potent mediator for these effects. The action of RES can provide cell survival and neuroplasticity, anti-inflammation, antioxidation, and autophagy via SIRT1 pathways. RES activates PPARγ to produce anti-inflammatory effects and NO to promote neurovascular coupling. It also initiates the generation of BDNF and synaptic-related proteins and suppresses BACE1 activity, which results in cell survival and neuroplasticity. Abbreviations: ac-NF-κB, acetylated nuclear factor kappa B; Akt, protein kinase B; BACE1, beta-site amyloid precursor protein cleaving enzyme 1; Bcl2, B-cell lymphoma 2; BDNF, brain-derived neurotrophic factor; CaMKII, calcium/calmodulin-dependent protein kinase II; CAT, catalase; DMT1, DNA methyltransferase 1; GABARR, γ-aminobutyric acid type A receptor; HO-1, heme oxygenase-1; IL-1β, interleukin-1 beta; IL-6, interleukin-6; Keap1, Kelch-like ECH-associated protein 1; LKB1, liver kinase B1; mt, mitochondria; miR-134, microRNA 134; mTOR, mammalian target of rapamycin; NF-κB, nuclear factor kappa B; NMDAR1, *N*-methyl-d-aspartate receptor subunit 1; NO, nitric oxide; NQO1, NAD(P)H quinone dehydrogenase 1; Nrf2, nuclear factor E2-related factor 2; PGC-1α, peroxisome proliferator-activated receptor γ coactivator 1-alpha; PPARγ, peroxisome proliferator activated receptor γ; PSD95, postsynaptic density 95; sAPPβ, soluble amyloid precursor protein beta-site; SIRT, sirtuin 1; SOD, superoxide dismutase; synGAP, synaptic Ras GTPase activating protein; TNFα, tumor necrosis factor alpha; TrkB, tyrosine kinase B. Created with BioRender/Mahidol University.

In conclusion, RES prevents neuronal cell death through a multifaceted approach that includes antioxidation, anti-apoptosis, anti-inflammation, and anti-amyloidogenic processing. Additionally, by improving cerebral blood flow, promoting autophagy, enhancing cell survival, and supporting neural plasticity, RES contributes to restoring and preserving cognitive function (Figure 5). These comprehensive neuroprotective properties highlight RES’s potential as a therapeutic agent for combating cognitive impairment and neurodegenerative conditions.

## 3 Effects of Resveratrol on Cognitive Impairment Restoration from Behavioral Studies

Cognitive performance refers to an individual’s ability to process, store, retrieve, and use information. This encompasses a range of cognitive domains, including sensation, perception, attention, memory, executive function, and language comprehension and production.^1^ To effectively address cognitive impairment, it is essential to thoroughly evaluate these cognitive domains, mainly focusing on working memory, attention, executive function, cognitive flexibility, and declarative memory.

Nevertheless, executive function is a cognitive process that the brain uses for planning and problem-solving. The executive function comprises of many aspects including working memory, attention, and cognitive flexibility.^117^ Consequently, the behavioral outcomes of these activities could represent as an indicator of executive function performance.

Several methods for conducting cognitive tests, each targeting different cognitive domains are described in the literature. These methods include the Morris water maze test (MWM), novel object recognition test,^118^ fear conditioning test (FCT), passive avoidance test (PAT), Barnes maze test (BMT), radial arm maze (RAM), and Y-Maze test (YMT). These assessments shed light on specific aspects of cognitive function and how treatments like RES impact them (Table 2).

**Table 2:** Effect of Resveratrol on Cognitive Function

model of study	dose	duration	behavioral test	behavioral results	outcomes	ref
Cerebrovascular aging model (6 months old male Sprague-Dawley rats)	1.25 mg/rat/day	5 months	NORT	↑ Time spent exploring the novel object	RES improved recognition memory.	(39)
	3,5,4′-trihydroxy-*trans*-stilbene (drinking water)			↓ Latency to the first exploration		
AD model: Hypertension-induced AD pathogenesis by using Angiotensin-II (10 weeks old male Wistar Kyoto rats)	10 mg/kg/day	14 days	MWM	↑ Time spent in the target quadrant	RES improved spatial learning and memory.	(43)
	3,5,4′-trihydroxy-*trans*-stilbene (intragastric)					
AD model: Intrahippocampal injection of Aβ_1–42_ (220–240 g, adult male Sprague-Dawley rats)	0.5, 1.25, 5, 22, 44 μmol 3,4,5′-trihydroxy-*trans*-stilbene (intrahippocampal)	Single dose	MWM	↓ Escape latency in RES-treated Aβ_1-42_ group	RES improved spatial learning and memory.	(44)
				↑ Time spent in the target quadrant in the RES-treated Aβ_1-42_ group	*However, RES did not show a memory-enhancing effect in the normal rats.	
				*↔ Escape latency and time in the target quadrant in RES-treated normal rats		
AD model: AlCl_3_ and D-galactose-induced sporadic AD mouse model (18–22 g, adult normal male Swiss mice)	40 mg/kg/day	10 weeks	NORT	↔ Sniffing frequency	RES did not affect recognition memory but improved the associative learning and memory.	(45)
	3,4′,5-trihydroxystilbene (intraperitoneal)		PAT	↑ Transfer latency		
AD model: a high-fat diet triggering AD progression in human APP and PSEN1 transgenic mouse model (5XFAD mouse)	120 mg/kg/day *trans*-resveratrol (intragastric)	16 weeks	NORT	↑ Discrimination index	RES recovered recognition memory.	(46)
AD model: APP/presenilin 1 double transgenic mouse model (18–22 g, 2.5 months old male Tg6799 mice)	60 mg/kg/day resveratrol (intragastric)	60 days	YMT	↑ Alternation percentage	RES improved spatial working memory.	(47)
			MWM	↓ Escape latency	RES improved spatial learning and memory.	
				↑ Target quadrant retention time		
AD model: Intrahippocampal injection of STZ (3 months old male C57BL/6 mice, *n* = 13/group)	25 mg/kg 3,5,4′-trihydroxy-*trans*-stilbene (intragastric)	5 weeks	MWM	↓ Escape latency in RES-treated STZ group	RES improved spatial learning and memory, as well as recognition memory.	(48)
			NORT	↑ Time spent in the target quadrant in the RES-treated STZ group		
				↑ Discrimination index		
Ischemic stroke model: Chronic cerebral hypoperfusion induced by bilateral common carotid artery occlusion (260–300 g, ∼10–18 weeks old male Sprague-Dawley rats)	50 mg/kg/day resveratrol (intragastric)	3, 6, and 9 weeks	MWM	↓ Escape latency	RES improved spatial learing and memory.	(54)
Ischemic stroke model: Middle cerebral ischemia/reperfusion (260–300 g, ∼10–18 weeks old Sprague-Dawley rats)	20 mg/kg/day *trans*-resveratrol (intraperitoneal)	7 days	MWM	↓ Escape latency	RES improved spatial learning and memory.	(55)
				↑ Number of crossings		
Ischemic stroke model: Bilateral common carotid artery occlusion (2 months old male Sprague-Dawley rats)	10 and 20 mg/kg/day 3,5,4′-trihydroxy-*trans*-stilbene (intraperitoneal)	4 weeks	MWM	↓ Escape latency	RES significantly improved spatial learning and memory.	(56)
				↑ Crossing frequency		
Ischemic stroke: Transient middle cerebral artery occlusion (2–3 months old male Sprague-Dawley rats, *n* = 5–7/group)	50 mg/kg 3,4,5′-trihydroxy-*trans*-stilbene (intragastric)	Single does	FCT	↑ Freezing time during the cued acquisition testing	RES restored associative learning and memory.	(63)
				↓ Freezing time during the extinction testing		
Chronic unpredictable mild stress model (180–200 g, ∼6–8 weeks old male Sprague-Dawley rats)	40 and 80 mg/kg/day 3,4′,5-trihydroxy-*trans*-stilbene (intraperitoneal)	4 weeks	MWM	↓ Escape latency on day 4 (80 mg/kg/day)	RES improved spatial learning and memory.	(69)
				↑ Time spent in the target quadrant (both doses)		
Chronic restraint stress-induced cognitive impairment model (8 weeks old male Wistar rats)	80 mg/kg/day resveratrol (intraperitoneal)	21 days	MWM	↓ Escape latency	RES improved spatial learning and memory, as well as recognition memory.	(70)
			NORT	↑ Time spent in the target quadrant		
				↑ Exploring time to the novel object during the retrieval phase		
				↑ Discrimination index		
Neuroinflammation model: LPS (8 weeks old male ICR mice)	5, 10, and 20 mg/kg/day 3,5,4′-trihydroxystilbene (intraperitoneal)	5 days	MWM	↓ Escape latency (10 and 20 mg/kg/day)	RES improved spatial learning and memory.	(73)
				↑ Time spent in the target quadrant (10 and 20 mg/kg/day)		
Neuroinflammation model: deep hypothermia (350–400 g, ∼12–14 weeks old male Sprague-Dawley rats)	30 mg/kg/day resveratrol (intravenous)	7 days	MWM	↓ Escape latency	RES improved spatial learning and memory.	(74)
				↑ Time spent in the target quadrant		
Neuroinflammation model: chemotherapeutic regimens (18–20 g, ∼6–9 weeks old female C57BL/6 mice)	50 and 100 mg/kg/day resveratrol (intragastric)	3 weeks	MWM	↓ Escape latency (100 mg/kg/day)	RES improved spatial learning and memory.	(75)
				↑ Time spent in the target quadrant (100 mg/kg/day)		
Cellular stress model: Heavy metal lead exposure (16 months old male C57BL/6 mice)	50 mg/kg/every other day	48 weeks	MWM	↓ Escape latency	RES improved spatial learning and memory.	(78)
	Resveratrol (intragastric)			↑ Time spent in the target quadrant		
Cellular stress model: Methamphetamine (18–22 g, ∼6–9 weeks old C57BL/6 mice)	10 and 100 mg/kg/day 3,5,4′-trihydroxy-*trans*-stilbene (intragastric)	17 days	NORT	↑ Discrimination index	RES significantly improved recognition memory.	(79)
Cellular stress model: pentylenetetrazole-induced epileptic seizure (250–400 g, ∼9–21 weeks old male Wistar rats)	40 mg/kg/day resveratrol (drinking water)	31 days	PAT	↔ Retention latency	RES did not show associative learning and memory restoration.	(80)
Cellular stress model: Paclitaxel (chemotherapy agent) (20–24 g, 6–8 weeks old male C57BL/6 mice, *n* = 10/group)	90 mmol/L/day resveratrol (intracerebroventricular)	7 days	MWM	↓ Escape latency	RES improved spatial learning and memory.	(81)
				↑ Platform crossing times		
Cellular stress model: Arsenic exposure (200–230 g, adult male Wistar rats, *n* = 8/group)	10 and 20 mg/kg/day 3,4′,5-trihydroxy-*trans*-stilbene (intraperitoneal)	21 days	MWM	↓ Escape latency	RES improved spatial learning and memory, as well as recognition memory.	(98)
			NORT	↑ Time spent in the target quadrant		
				↑ Discrimination index		
Cellular stress model: Manganese exposure (23–25 g, 6 weeks old adult Kunming mice, male:female ratio = 1:1)	30 mg/kg/day	6 weeks	MWM	↓ Escape latency	RES improved spatial learning and memory.	(99)
	3,4′,5-trihydroxystilbene (intragastric)			↑ Time spent in the target quadrant		
POCD model (21 months old male Sprague-Dawley rats)	10 mg/kg/day resveratrol (intraperitoneal)	7 days	MWM	↓ Escape latency	Pretreatment with RES improved spatial learning and memory, as well as associative learning and memory.	(101)
			FCT	↑ Time spent in the target quadrant		
				↑ Freezing time during the context test		
				↔ Freezing time during the tone test		
POCD model (21 months old male Sprague-Dawley rats)	10 mg/kg/day	15 days	MWM	↔ Escape latency compared to the control + RES group	RES improved spatial learning and memory.	(102)
	3,4,5-trihydroxystilbene (intraperitoneal)			↔ Platform crossing time compared to the control + RES group		
POCD model (18 months old male C57BL/6 mice)	100 mg/kg/day resveratrol (intraperitoneal)	7 days	FCT	↑ Freezing time in both context and tone tests	Pretreatment with RES restored associative learning and memory.	(103)
POCD model (18 months old male C57BL/6 mice)	50 mg/kg/day resveratrol (intraperitoneal)	7 days	BMT	↓ Time to identify target box	RES improved spatial learning and memory, as well as associative learning and memory.	(104)
			FCT	↑ Freezing time in both context and tone tests		
POCD model (16 months old male C57BL/6 mice)	10 mg/kg/day resveratrol (intraperitoneal)	5 days	MWM	↓ Escape latency	RES improved spatial learning and memory.	(115)
				↑ Time spent in the target quadrant		
Metabolic disease model: Diabetes mellitus type II induced by streptozotocin and a high-fat diet (8 weeks old male ICR mice)	30 mg/kg/every other day resveratrol (intragastric)	4 months	MWM	↓ Escape latency	RES improved spatial learning and memory.	(85)
				↑ Time in the target quadrant		
				↑ Times across platform		
Metabolic disease model: Obesity induced by a high-fat diet (15 months old male C57BL/6 mice)	100 mg/kg/day resveratrol (intraperitoneal)	7 days	FCT	↑ Freezing time	RES restored associative learning and memory.	(90)
Metabolic disease model: Diabetic model induced by streptozotocin (250–300 g, adult male Wistar rats)	20 mg/kg/day resveratrol (intraperitoneal)	4 weeks	MWM	↓ Escape latency	RES improved spatial learning and memory, as well as associative learning and memory.	(109)
			PAT	↑ Time spent in the target quadrant		
				↑ Retention latency		
Metabolic disease model: Fructose feeding model (6–12 months old Wistar rats)	2 mg/kg/day natural resveratrol (intragastric)	16 weeks	BMT	↓ Finding target distance	RES improved spatial learning and memory.	(110)
				↑ Finding target speed		
Metabolic disease model: NAFLD model induced by a high-fat diet (2 months old male Sprague-Dawley rats)	15 mg/kg/day 3,5,4′-trihydroxy-*trans*-stilbene (intragastric)	4 weeks	MWM	↓ Escape latency	RES improved spatial learning and memory.	(111)
				↑ Target quadrant retention time		
Metabolic disease model: Diabetes mellitus type II induced by streptozotocin and a high-fat diet (22–25 g, 8 weeks old, male C57BL/6 mice, *n* = 12/group)	5 mg/kg/day	8 weeks	NORT	↑ Exploring time to the new object during the test phase	RES improved recogtion memory, spatial short-term memory, as well as associative learning and memory.	(112)
	3,4′,5-trihydroxy-*trans*-stilbene (drinking water)		YMT	↑ Discrimination index		
			FCT	↑ Exploring time to the new arm during the test phase		
				↑ Stagnation level (freezing time)		
Metabolic disease model: Diabetes mellitus type II induced by streptozotocin and a high-fat diet (125–150 g, adult male Albino Wistar rat, *n* = 10/group)	20 mg/kg/day resveratrol (intraperitoneal)	4 weeks	MWM	↓ Escape latency	RES improved spatial learning and memory.	(113)
				↑ Time in the target quadrant		
Metabolic disease model: Diabetes mellitus type II induced by streptozotocin and a high-fat diet (180–220 g,12–14 weeks old, adult male Sprague-Dawley rats, *n* = 42/group)	50, 100 mg/kg/day resveratrol (intraperitoneal)	4 weeks	NORT	↑ Exploring time to the novel object during the retention phase	RES improved recognition memory, as well as spatial learning and memory.	(114)
			RAM	↓ Total number of errors		
				↓ Reference and working memory errors		

### 3.1 Assessment of Declarative Learning and Memory

Declarative memory, also known as explicit memory, involves recalling events and facts; episodic memory specifically refers to the recollection of personal experiences. Within this category, spatial and recognition memories are subtypes where animals must remember the relationships between objects, places, and events to complete tasks successfully.

To assess these memories, tests like MWM, YMT, BMT, RAM, and NORT are frequently employed. These tests can assess executive function because animals use their prefrontal cortex and hippocampal region to determine how to move toward a goal-directed strategy to achieve a task.^119^

*BMT* evaluates spatial memory. In this task, animals use visual cues to find an escape hole located on an open platform. The BMT assesses the animal’s ability to learn and remember the escape hole’s location, which indicates its spatial memory capabilities.^119^

*RAM* is another commonly used spatial memory test that assesses an animal’s ability to associate specific arms of the maze with rewards (typically food). Animals with better spatial memory can recall which arms have been visited and rewarded, avoiding re-entry into those arms. This task is beneficial for evaluating both reference and working memory.^119^

*YMT* is widely used to elucidate spatial short-term memory, requiring the involvement of the hippocampus and prefrontal cortex. During this test, animals are allowed to explore the Y-shaped maze. A lower percentage of entries into previously visited arms indicates better short-term memory, as healthy animals tend to explore new, unvisited arms.^120^

*NORT* assesses recognition memory, relying on the hippocampus and prefrontal cortex for object-in-place memorization. In NORT, animals are exposed to both familiar and novel objects. The premise of this test is that animals will spend more time exploring novel objects than familiar ones, reflecting their recognition memory capabilities.^121^

In this review, we summarize that the administration of RES enhances spatial learning and memory, as evidenced by improved performance in the MWM, BMT, and RAM. Studies have shown that RES-treated animals exhibit shorter escape latencies and increased time spent in target areas or correct arms, demonstrating superior spatial memory and learning.^43,44,47,48,54−56,69,75,78^ Improvements in spatial working memory have also been observed in the YMT, where RES-treated animals show increased spontaneous alternation behavior, indicating better cognitive flexibility and short-term memory.^47,112^

While most research indicates that RES improves recognition memory, as evidenced by enhanced performance in NORT,^39,46,48,70,79,98,112,114^ some conflicting findings exist. A study by Labban et al.^45^ using a sporadic Alzheimer’s disease mouse model induced by AlCl_3_ and D-galactose reported contrary results. This highlights the variability in response to RES treatment, possibly due to differences in experimental models or treatment protocols.

Behavioral studies suggest that RES significantly enhances declarative memory, including spatial and recognition memory, and executive function. These improvements are likely due to RES’s neuroprotective effects, such as reducing oxidative stress and inflammation, promoting neurogenesis, and enhancing synaptic plasticity. Such findings underscore the potential of RES as a therapeutic agent for mitigating cognitive impairments associated with neurodegenerative diseases.

### 3.2 Assessment of Associative Learning

Associative learning is a fundamental process through which organisms learn to associate two unrelated stimuli.^122^ This type of learning is essential for adaptation to the environment and is classified under nondeclarative memory (implicit memory). Two prominent examples of associative learning tests that evaluate aversive behavior are the fear conditioning test (FCT) and the passive avoidance test (PAT).

FCT operates on the principle that animals learn to exhibit a fear response (e.g., freezing) to a neutral stimulus (e.g., tone) when it is paired with an aversive stimulus (e.g., electric foot shock). Successful associative learning is indicated by the animal’s freezing behavior upon hearing the tone, demonstrating recognition of the association between the tone and the foot shock. This form of learning involves the hippocampus, amygdala, and prefrontal cortex.^123,124^

PAT evaluates the animal’s ability to avoid a compartment where an aversive stimulus was previously delivered. This avoidance behavior reflects associative learning, as the animal must remember and avoid the location associated with the unpleasant stimulus. Like the FCT, this task also requires the functional integration of the hippocampus, amygdala, and prefrontal cortex.^123,124^

A literature review reveals that injured animals often exhibit impaired aversive behaviors, characterized by reduced freezing times in response to the tone or a preference for entering the area where the aversive stimulus was previously administered. However, these deficits were ameliorated following treatment with RES. Studies have shown that RES-treated animals regained normal aversive behaviors, evidenced by increased freezing times and avoidance of the aversive area.^45,63,90,103,104,109,112^ Conversely, some research has indicated that RES does not affect associative memory, highlighting variability in outcomes potentially due to differences in animal models, dosages, and treatment durations.^80,101^

Based on results from these cognitive assessments, it is suggested that RES can restore cognitive function. The observed improvements in behavioral performance imply that RES may modulate the hippocampus and prefrontal cortex activity, which aligns with findings from molecular studies. Specifically, RES is known to exert neuroprotective effects by reducing oxidative stress, inflammation, and apoptosis and enhancing synaptic plasticity and neurogenesis.

Despite the substantial focus on declarative, working, and associative memory in existing studies, there is a notable gap in understanding the effects of RES on cognitive flexibility. Cognitive flexibility is a component of executive functions that adapt responses to new or changing situations. It is often impaired in early stage AD.^125^ The same tests used to evaluate declarative memory can also assess cognitive flexibility by incorporating a reversal learning phase. In this phase, animals must disregard previous learning, adapt to new rules or goals, and develop new task-specific strategies, necessitating higher-order cognitive functions primarily mediated by the prefrontal cortex.^126^

Evaluating cognitive flexibility through reversal learning provides a more comprehensive understanding of cognitive function and RES’s therapeutic potential. Therefore, incorporating assessments of cognitive flexibility in future studies is recommended to elucidate the full spectrum of RES’s cognitive benefits.

## 4 Pharmacokinetic Challenges in Translating Preclinical Findings to Clinical Outcomes

In human clinical trials, RES has shown promise in improving cognitive functions in older adults. Witte et al. in 2014 conducted a randomized, double-blind, placebo-controlled study involving 46 elderly individuals. Participants received either RES supplementation or a placebo for 26 weeks. The RES group exhibited significant improvements in memory performance and increased functional connectivity within the hippocampus, as measured by functional MRI. Additionally, RES improves glucose metabolism, which is crucial for optimal brain function.^127^

In addition, a case-control study enrolled 30 selected patients who had been clinically diagnosed with moderate to mild AD. The findings suggest that patients receiving *trans*-RES may have a beneficial effect in slowing AD progression compared to the placebo group. The improvement in cognitive scores, alongside favorable biomarker and neuroimaging results, supports the potential of *trans*-RES as a therapeutic agent for AD.^128^

Another notable study by Turner et al. in 2015 explored the effects of RES on cognitive decline in patients with mild to moderate AD over 52 weeks. While the study found that RES was well-tolerated and had some beneficial effects on biomarkers associated with AD, it did not result in significant cognitive improvements compared to the placebo group. The primary cognitive end points, measured by standardized tests such as the AD Assessment Scale–cognitive (ADAS-Cog), did not show statistically significant differences between the RES and placebo groups.^129^

Despite RES’s promising potential for cognitive impairment, not all studies have shown positive results. Some studies reported negative or inconclusive outcomes regarding the use of RES for cognitive impairment in humans.

Although numerous preclinical studies have demonstrated the neuroprotective and cognitive-enhancing effects of RES, the results of clinical studies have been less encouraging. One of the most significant challenges in translating these findings from animal models to humans is the pharmacokinetic differences between species. These differences profoundly impact the bioavailability and therapeutic efficacy of RES.

Pharmacokinetic differences between rats and humans, particularly in the context of gut metabolism within the enterohepatic circulation, have been reported. Specifically, the two species’ metabolic capacities for glucuronidation and sulfation, crucial processes for RES metabolism, are significantly different. It has been observed that rats have a lower capacity for glucuronidation and sulfation in the gut than humans.^127^ This reduced metabolic capacity in rats leads to higher circulating concentrations of RES, thereby enhancing its bioavailability and resulting in more pronounced effects in preclinical studies.

In humans, the higher metabolic capacity for glucuronidation and sulfation reduces the bioavailability of RES, as a substantial portion of the compound is metabolized before reaching systemic circulation. This discrepancy contributes to the lower efficacy observed in clinical studies. Physiologically based pharmacokinetic (PBPK) modeling supports this assumption, indicating that the pharmacokinetic profiles of RES differ significantly between rats and humans.^130^ These models demonstrate that the extensive metabolism of RES in the human gut reduces its systemic exposure, thereby diminishing its therapeutic potential.

Understanding these pharmacokinetic disparities is crucial for developing strategies to enhance RES’s clinical efficacy. Modifying RES formulations to improve bioavailability, coadministration with metabolic inhibitors, or the development of analogs with better pharmacokinetic profiles could potentially bridge the gap between preclinical and clinical outcomes.

In summary, while preclinical studies provide valuable insights into the neuroprotective effects of RES, translating these findings to clinical applications remains challenging due to significant pharmacokinetic differences between species. Addressing these challenges through innovative pharmacological strategies is essential for realizing the therapeutic potential of RES in human cognitive health.

## 5 Adverse Effects of Resveratrol

In addition to effectiveness data, RES safety data have been reported. Clinical toxicity data has been consistent with findings from *in vitro* and *in vivo* studies RES demonstrates a hermetic dose–response effect, where low doses offer protective benefits (e.g., antioxidant, anti-inflammatory) while high doses can trigger toxic responses, such as increased oxidative stress and organ damage, particularly after prolonged exposure.^131^ Fortunately, RES is widely accepted to be safe at therapeutic doses. The potential adverse effects and some points of concern of RES including drug interaction and pharmacokinetic challenges are summarized.^132−136^

### 5.1 *In Vitro* Studies

#### Cytotoxicity

*In vitro* studies reveal that RES exhibits a dose-dependent cytotoxicity in various cell types, including cancer and normal cells. While it is primarily considered protective and anticancer at lower concentrations, it may cause cell death at higher doses through mechanisms such as apoptosis and necrosis.

#### Genotoxicity

Some studies indicate that RES can cause DNA damage, chromosomal aberrations, and interfere with normal cell cycle regulation, particularly at higher concentrations or with prolonged exposure.

#### Membrane Interaction

RES can alter membrane fluidity and permeability, affecting cellular function. At higher doses, its interaction with cellular membranes may disrupt normal membrane integrity and signaling processes.

### 5.2 *In Vivo* Studies (Animal Studies)

#### Hepatotoxicity

High doses of RES in animal models have led to liver damage and oxidative stress. Some mice developed signs of liver injury with elevated markers of liver dysfunction.

#### Nephrotoxicity

RES at high doses has been linked to kidney toxicity in specific animal models, with histopathological changes in kidney tissues observed.

#### Reproductive Toxicity

Animal studies suggest potential reproductive toxicity, with high doses of RES leading to reduced fertility, sperm abnormalities, and impaired reproductive function in both male and female rodents.

#### Gastrointestinal Toxicity

Large doses of RES have been associated with diarrhea, weight loss, and gastrointestinal irritation in some animal models.

### 5.3 Human (Clinical) Studies

#### Tolerability and Safety

In human clinical studies, RES appears well-tolerated at lower doses (up to 1 g/day) for short periods. It is associated with mild side effects, primarily gastrointestinal disturbances such as nausea, diarrhea, and abdominal pain.

#### High-Dose Toxicity

Some clinical studies using doses higher than 1 g/day report increased side effects, including gastrointestinal discomfort and mild liver enzyme elevation. However, severe adverse effects in humans are rare, and most reported side effects are transient and reversible.

#### Long-Term Effects

The long-term safety of high-dose RES in humans remains unclear due to limited long-term clinical data.

#### Drug Interaction

RES at a dosage of 1000 mg/day or above has been shown to inhibit cytochrome P450 isoenzymes such as CYP3A4, CYP2C9, and CYP2D6 and to activate CYP1A2, which can cause interactions with other drugs. Therefore, orally administered high doses (more than 1000 mg/day) of RES indicate variations in the pharmacokinetics of coadministered drugs.

#### Pharmacokinetic Challenges

RES’s systemic benefits in humans are limited by its low bioavailability and rapid metabolism since only a small fraction of the orally administered compound reaches circulation in its active form. Thus, improving the limited bioavailability and stability will reduce the requirement for high RES doses and lower adverse effects.

Conclusively, based on current clinical and preclinical studies, RES is generally considered safe at therapeutic doses. However, high doses, particularly in animal models, reveal potential toxicity, such as liver and kidney damage. Human clinical data indicate well tolerability at lower doses (up to 1,000 mg/day), but the long-term safety of high doses remains under investigation. Comprehensive long-term studies are needed to establish the safety of chronic high-dose RES use. In particular, the optimal dose capable of maximizing health benefits without raising toxicity remains an area of extensive research. In addition, the characteristics of the patients and the duration of RES supplementation should be considered. However, by addressing pharmacokinetic limitations, therapeutic benefits could be maximized while potential toxicity could be minimized. Furthermore, data on the interactions of RES when combined with other therapies are insufficient.

## 6 Discussion and Conclusions

The activities of RES at the cellular level are well-correlated with its effects at the behavioral level. Data from various animal models suggest that RES exerts its neuroprotective effects through multiple molecular mechanisms, including anti-inflammation, antioxidation, anti-apoptosis, and neurotrophic properties. These underlying mechanisms support the role of RES in cognitive restoration observed in behavioral studies.

However, the existing behavioral studies have a significant limitation: they lack data on the cognitive flexibility domain, which involves the ability to adapt to new and changing situations. Cognitive flexibility is crucial for everyday functioning and is often impaired in neurodegenerative diseases. A well-organized behavioral study design focusing on the cognitive flexibility domain is recommended to address this gap. Such studies would provide a more comprehensive understanding of RES’s impact on different cognitive functions and offer insights into its potential therapeutic benefits.

Moreover, there are notable differences in the pharmacokinetics of RES between rats and humans. This poses a challenge in translating animal study findings to clinical settings. In humans, RES undergoes rapid metabolism in the gut and liver, which can limit its bioavailability and efficacy. To maximize RES activity in clinical studies, the development of RES formulations that delay its metabolism during enterohepatic circulation should be prioritized. Such formulations would enhance the bioavailability of RES, ensuring higher and more sustained levels of the compound in the bloodstream and, consequently, more pronounced therapeutic effects.

For safety data, RES is considered safe at low doses *in vitro* and *in vivo* studies and generally appears to be well tolerated at therapeutic doses (up to 1,000 mg/day) in clinical studies. However, the long-term safety of high doses of RES should be further investigated.

In conclusion, while preclinical studies demonstrate the potential of RES in neuroprotection and cognitive restoration, further research is necessary to explore its effects on cognitive flexibility. Additionally, optimizing RES formulations for better bioavailability is crucial for translating these findings into effective clinical treatments for cognitive impairment and neurodegenerative diseases. Long-term studies to examine the ideal dosage to optimize health benefits without increasing adverse effects for continuous use are considered.
